# Molecular developmental mechanism in polypterid fish provides insight into the origin of vertebrate lungs

**DOI:** 10.1038/srep30580

**Published:** 2016-07-28

**Authors:** Norifumi Tatsumi, Ritsuko Kobayashi, Tohru Yano, Masatsugu Noda, Koji Fujimura, Norihiro Okada, Masataka Okabe

**Affiliations:** 1 Department of Anatomy, The Jikei University School of Medicine, 3-25-8 Nishi-Shimbashi, Minato-ku, Tokyo 105-8461, Japan; 2 Department of Environmental Science, Niigata University, 8050 Ikarashi 2-no-cho, Nishi-ku, Niigata, Niigata 950-2181, Japan; 3 Department of Life Sciences, National Cheng Kung University, Tainan 701, Taiwan; 4 Foundation for Advancement of International Science, Tsukuba 305-0821, Japan

## Abstract

The lung is an important organ for air breathing in tetrapods and originated well before the terrestrialization of vertebrates. Therefore, to better understand lung evolution, we investigated lung development in the extant basal actinopterygian fish Senegal bichir (*Polypterus senegalus*). First, we histologically confirmed that lung development in this species is very similar to that of tetrapods. We also found that the mesenchymal expression patterns of three genes that are known to play important roles in early lung development in tetrapods (*Fgf10, Tbx4, and Tbx5*) were quite similar to those of tetrapods. Moreover, we found a *Tbx4* core lung mesenchyme-specific enhancer (C-LME) in the genomes of bichir and coelacanth (*Latimeria chalumnae*) and experimentally confirmed that these were functional in tetrapods. These findings provide the first molecular evidence that the developmental program for lung was already established in the common ancestor of actinopterygians and sarcopterygians.

Tetrapods use lungs for respiration, which deliver oxygen from the air into the body and remove carbon dioxide from the body. Lung development has been well studied in tetrapods^1^, where it has been shown that signals from the lateral plate mesoderm first stimulate the foregut endoderm to form a primary lung bud from the ventral part. This lung bud then elongates and branches into two buds to form the lobes of the lungs. Lungs are not only found in tetrapods but also in the sarcopterygian lungfish, which also use these for respiration^2^. Moreover, extant coelacanths (*Latimeria*), which belong to the sister group of lungfish and tetrapods, possess a tubular “fat-filled organ” arising from the ventral part of the esophagus. This organ is believed as a “gas bladder” that is used to control buoyancy^3^. It is thought that fossil coelacanths possessed lungs, and therefore, it was long believed that coelacanths lost or transformed these lungs into this fat-filled organ^4^. However, it has recently been shown that at the embryo stage, coelacanths possess a single lung bud in addition to this fat-filled organ^5^. Furthermore, it has been found that a *Tbx4* lung mesenchyme-specific enhancer (LME), which was first identified in the mouse (*Mus musculus*) genome and regulates the expression of the *Tbx4* gene in the lung mesenchyme from early development^6^,^7^, is well-conserved in the coelacanth (*Latimeria chalumnae*) genome^8^,^9^. Taken together, these findings suggest that coelacanths possess the homologous lung similar to other members of the sarcopterygian crown group and that the *Tbx4* lung enhancer (and lungs) may be present in the last common ancestor of crown sarcopterygians.

The lung developments of lobe-finned fish (lungfish and coelacanths) are ideal candidates for understanding vertebrate lung evolution. However, it is difficult to obtain embryos from these species and the whole genome sequence of the lungfish is currently unavailable due to its extremely large size^10^, which makes it difficult to read the sequence. Consequently, polypteriformes (bichirs and reedfish) may be better candidates for investigating the evolution of the vertebrate lung. Because the family Polypteridae (Actinopterygii) also possess lungs (gas-filled organs on the ventral side)^2^,^11^,^12^,^13^ for air breathing, it is believed that the common ancestor of sarcopterygians and actinopterygians already possessed lungs^2^,^3^,^12^,^14^,^15^. Polypterids are now recognized as the earliest diverging lineage of the Actinopterygii (ray-finned fish)^16^,^17^,^18^, and display many primitive characters that are not found in other living actinopterygians^19^,^20^,^21^,^22^,^23^. A histological analysis of lung development in Senegal bichir (*Polypterus senegalus*) indicated that this species has a similar developmental mechanism to tetrapods^11^. Although it is difficult to obtain bichir embryos, it is possible to breed these fish under laboratory conditions^24^,^25^. Moreover, several molecular biological studies using bichir embryos have been published in recent years^25^,^26^.

In this study, we investigated the lung development of *P. senegalus* in an attempt to further elucidate the evolution of vertebrate lungs. We histologically examined lung development in bichir and then investigated the expression patterns of four genes that are known to play an important role in early lung development in mouse and chicken (*Gallus gallus*), demonstrating strong similarities between the bichir and tetrapods. We also found a *Tbx4* core lung mesenchyme-specific enhancer (C-LME) in the genome of *P. senegalus* as well as the coelacanth *L. chalumnae* and experimentally confirmed that these are functional in chicken (tetrapod) embryos. Thus, our findings indicate that the molecular mechanism for lung development in tetrapods is conserved in bichir and strongly suggest that lungs were already present in the common ancestor of actinopterygians and sarcopterygians.

## Results

### Lung development

**Figure 1:**
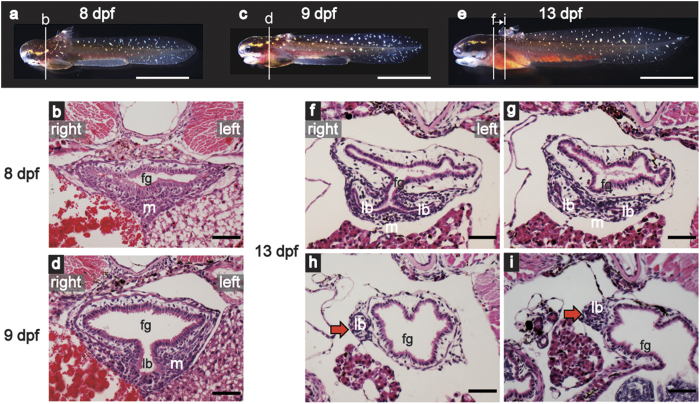
Lung development in *Polypterus senegalus*. Specimens at (**a**) 8 days post fertilization (dpf), (**c**) 9 dpf, and (**e**) 13 dpf; and sections at (**b**) 8 dpf, (**d**) 9 dpf, and (**f**–**i**) 13 dpf stained with hematoxylin and eosin. Red arrows indicate the right lung bud. Scale bars, 100 μm. fg, foregut; lb, lung bud; m, mesenchyme.

To observe lung development in *P. senegalus* in more detail, we made paraffin sections of larvae at several stages. At 8 days post fertilization (dpf, Fig. 1a), the endodermal foregut was still a tubular structure that was surrounded by mesenchymal cells, which were denser in the ventral part of the foregut than in the dorsal part (Fig. 1b). At 9 dpf (Fig. 1c), a primary lung bud had arisen from the foregut tube (Fig. 1d), which closely resembled that observed during tetrapod lung development. At 13 dpf (Fig. 1e), the primary lung bud had already divided into the left and right buds (Fig. 1f,g). It is known that the lungs of *Polypterus* are asymmetrical^13^, with the right lung being longer than the left lung, and caudal serial sections at 13 dpf supported this, showing that the right lung extended more posteriorly than the left lung (red arrows on Fig. 1h,i). These results demonstrate that the primary lung bud begins to develop after hatching.

### Gene expression patterns

**Figure 2:**
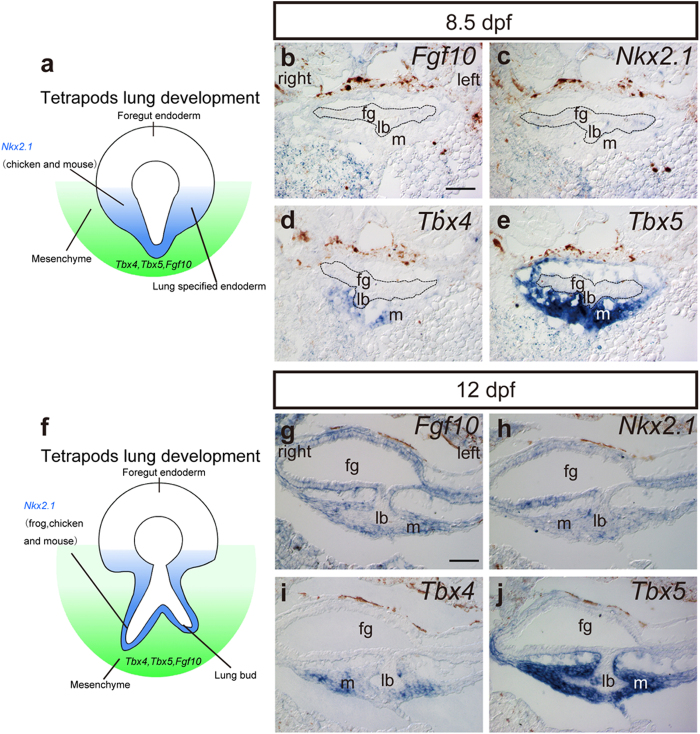
Gene expression patterns of *Polypterus senegalus* embryos. Gene expression patterns of schematic images in tetrapods and of *P. senegalus* at (**a**–**e**) 8.5 days post fertilization (dpf) and (**f**–**j**) 12 dpf for the genes *Fgf10 (***b**,**g**), *Nkx2.1* (**c**,**h**), *Tbx4* (**d**,**i**), and *Tbx5* (**e**,**j**). Dotted lines in (**b**–**e**) indicate the foregut endoderm and lung bud. Scale bars, 100 μm. fg, foregut; lb, lung bud; m, mesenchyme.

To clarify the similarity in early lung development between bichir and tetrapods at the molecular level, we investigated the expression patterns of four genes (*Nkx2.1*, *Fgf10, Tbx4* and *Tbx5*) that are known to play an important role in early lung development in mouse and chicken^27^,^28^,^29^,^30^,^31^,^32^ (Fig. 2a,f). We cloned the orthologs of these four genes and compared the amino acid sequences to confirm their similarities across several species (see Supplementary Fig. S1). We then used RNA probes constructed from the clones of the four genes and performed *in situ* hybridization to observe the expression patterns during lung development in bichir (Fig. 2b–e,g–j).

At 8.5 dpf, no expression of *Fgf10* and *Nkx2.1* was observed (Fig. 2b,c); however, expression of *Tbx4* and *Tbx5* was detected at the mesenchyme of the developing lung bud, with *Tbx4* being weakly expressed in the ventral part (Fig. 2d) and *Tbx5* being strongly expressed in the mesenchyme (Fig. 2e). At 12 dpf, the expression of *Fgf10* was also detected in the surrounding mesenchyme (Fig. 2g), while *Nkx2.1* had very weak expression in the foregut and lung bud (Fig. 2h). At this time, *Tbx4* expression was observed in the more ventral part of the mesenchyme, at the pointed tips of the left and right branched buds (Fig. 2i), whereas *Tbx5* was strongly detected in the mesenchyme around the entire lung bud (Fig. 2j).

### Core lung mesenchyme-specific enhancer in the *P. senegalus* genome

The patterns of gene expression present in *P. senegalus* were similar to those of tetrapods suggest that they may be driven by the same regulatory mechanism. Therefore, we investigated whether bichir has conserved regulatory elements for lung development in its genome. As the mouse lung mesenchyme-specific enhancer (LME) of *Tbx4* has previously been identified in several species^6^,^7^, including coelacanths^8^,^9^, we focused on the regulatory elements of this gene.

**Figure 3:**
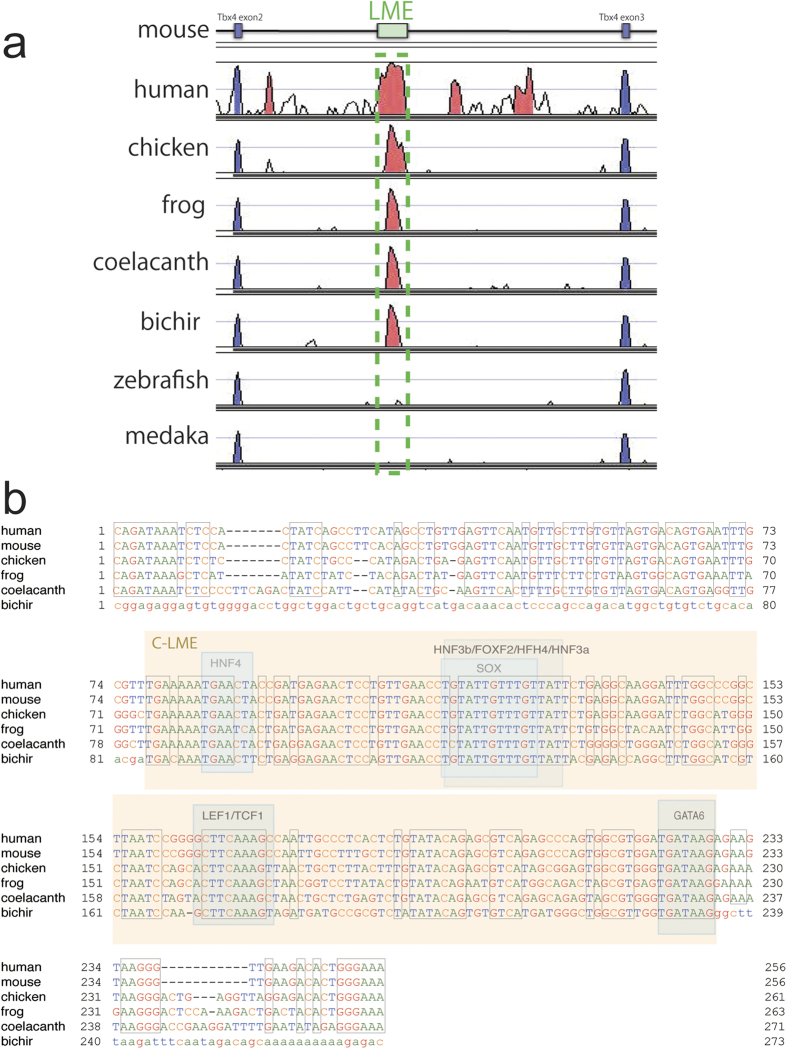
The mouse lung mesenchyme-specific enhancer (LME) of the *Tbx4* gene. (**a**) VISTA plots comparing the mouse LME sequences of the *Tbx4* gene in human (*Homo sapiens*), chicken (*Gallus gallus*), frog (*Xenopus tropicalis*), coelacanth (*Latimeria chalumnae*), bichir (*Polypterus senegalus*), zebrafish (*Danio rerio*), and medaka (*Oryzias latipes*) with that in mouse (*Mus musculus*). The green dashed box indicates the conserved region in the LME. (**b**) Multiple sequence alignment of the conserved region among human, mouse, chicken, frog, coelacanth and bichir. A core lung mesenchyme-specific enhancer (C-LME) of 150 bp was found in bichir as well as the sarcopterygians, as shown in orange. The predicted binding sites of the transcription factors are indicated by blue boxes.

VISTA plots (Fig. 3a) showed that a conserved region corresponding to the LME was found in each of the genomes of the sarcopterygians, but not in zebrafish (*Danio rerio*) or medaka (*Oryzias latipes*), both of which are actinopterygians. However, interestingly, we found that *P. senegalus* also has the conserved region, despite this species also being included in the actinopterygians. The multiple sequence alignment showed that the 150-bp region was conserved in the genomes of *P. senegalus* and the sarcopterygians, consistent with the possession of lungs of these species (Fig. 3b). We named this conserved sequence a “core lung mesenchyme-specific enhancer” (C-LME).

### Activity of the C-LME in chicken embryos

**Figure 4:**
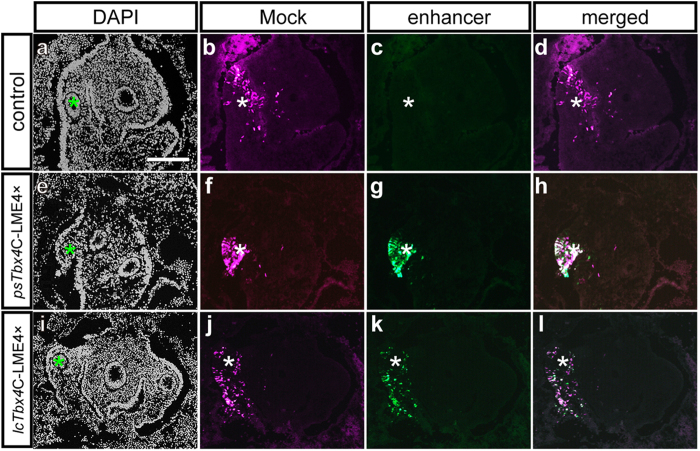
Functional assay for the core lung mesenchyme-specific enhancer of the *Tbx4* gene. Various vectors were introduced into the right-hand side of the chicken lung mesenchyme: (**a**–**d**) the ptk-EGFP vector alone as a control, (**e**–**h**) *psTbx4*C-LME4×, and (**i**–**l**) *lcTbx4*C-LME4×. pCAGGS-Kusabira-orange was co-introduced to monitor the plasmid-positive area (**b**,**f**,**j**). The nuclei were stained with 4′,6-diamidino-2-phenylindole (DAPI) (**a**,**e**,**i**). No expression was observed in the control (**c**), whereas enhanced green fluorescent protein (EGFP) expression from *psTbx4*C-LME4× and *lcTbx4*C-LME4× was detected in the lung mesenchyme (**g**,**k**). The signals from Kusabira-orange were merged with the EGFP expression (**d**,**h**,**l**). Asterisks indicate the right lung bud. Scale bars, 200 μm.

To verify whether the C-LME is functional, we tested its expression activity in chicken embryos (Fig. 4a–l). Four tandemly repeated C-LMEs of *P. senegalus* were cloned into the ptk-EGFP vector. The plasmids were then introduced into the prospective lung mesenchyme of chicken embryos [Hamburger–Hamilton (HH)^33^ stage 12]. The embryos were observed 48 h later, using Kusabira-orange to monitor the plasmid-positive area (Fig. 4b,f,j). No expression was observed in the control embryos (Fig. 4c; *n* = 5), but enhanced green fluorescent protein (EGFP) expression from *psTbx4*C-LME 4× was detected in the lung mesenchyme (Fig. 4g; *n* = 6), demonstrating that the C-LME of *P. senegalus* is functional for expressing the *EGFP* gene in the tetrapod.

We found C-LME sequences were highly conserved between bichir and coelacanth *Latimeria chalumnae*. Therefore, we also performed the same experiments using the C-LME from *L. chalumnae* genome (*lcTbx4* C-LME 4× Fig. 4k; *n* = 7), which provided the same results as that of bichir.

## Discussion

It is now believed that lungs were not acquired during the terrestrialization of vertebrates, but rather originated in a common ancestor of the actinopterygians and sarcopterygians^2^,^3^,^12^,^14^,^15^. Evidences from the fossil record and phylogenies based on morphological characteristics indicate that the last common ancestor of actinopterygians and sarcopterygians lived during the late Silurian^34^,^35^,^36^,^37^,^38^,^39^,^40^. Our molecular data from *P. senegalus* strongly supported the presence of lungs at the osteichthyan crown group node at least 423 million years ago.

Polypterids diverged from the common ancestor of the actinopterygians approximately 380 million years ago in the Devonian period^16^,^17^,^18^ and consequently, possess many primitive characters that are not observed in other actinopterygians^19^,^22^. One particular characteristic of this family is the presence of lungs, which developed from the ventral side of the foregut endoderm. Our histological observation of lung development in *P. senegalus* showed that the lungs of this species develop in a similar manner as those of tetrapods and compensated for that of Kerr^11^, who shows the development of this species based on the limited specimens.

Investigation of the expression patterns of four genes that are known to play an important role in early lung development in mouse and chicken showed that three of these (*Fgf10*, *Tbx4*, and *Tbx5*) had similar expression patterns in bichir. *Tbx4* and *Tbx5* are transcription factors that are involved in the induction and elongation of the tetrapod lung bud^30^ and were expressed in the mesenchyme of both the foregut and lung bud in bichir; *Fgf* signaling is known to be important for the elongation and growth of the tetrapod lung bud^32^,^41^, confirming that *Fgf10* expression was observed in the mesenchyme of the elongating lung bud in bichir. However, the expression pattern of *Nkx2.1* in bichir appeared to be different from that of mouse and chicken, where it is expressed during very early development^27^,^28^ and is involved in the specification of the foregut endoderm into the lungs. In contrast, no expression of this gene was detected in bichir at 8.5 dpf. Similarly in *Xenopus*, it appears that *Nkx2.1* does not play this early developmental role, only being detectable from the start of lung bud development^42^. This result suggests that the timing of *Nkx2.1* expression during early lung development is a specialization of amniotes with *Xenopus* and *Polypterus* both retaining the primitive osteichthyan condition. Thus, overall, the gene expression patterns during bichir lung development suggest that the molecular mechanism for lung development in bichir is similar to that of tetrapods.

To further investigate this idea, we examined whether polypterids possesses conserved regulatory elements for lung development in its genome. We found that the C-LME of the *Tbx4* gene was conserved in the genomes of *P. senegalus* and the sarcopterygians (coelacanth and tetrapods). Furthermore, functional experiments showed that the C-LMEs of both bichir and coelacanth act as enhancers, driving the expression of EGFP in the tetrapod lung mesenchyme in our studies. Thus, these results suggest that the polypterids have regulatory mechanisms of lung development that has been conserved in the sarcopterygians. The sarcopterygii and actinopterygii diverged more than 425 million years ago^36^,^38^,^39^, and yet, bichir has conserved the C-LME. This suggests that there are some important regulatory elements in C-LME for lung development. The bichir C-LME sequence had 44% and 66% similarities with mouse and coelacanth sequences, respectively. Coelacanth C-LME was much more similar to that in tetrapods than that in *P. senegalus*, but some regions were well conserved through the sarcopterygians and bichir. We predicted that C-LME would contain several transcription factor binding sites and found well-conserved regions indicating such sites. In total, five transcription factor sites were found [HNF4, HNF3 (and related binding sites FOXF2, etc.), SOX, TCF1/LEF1, and GATA6], all of which are known to contribute to lung development in some way (blue boxes in Fig. 3b). Among these transcription factors, it has been reported that *Foxf*2 and *Gata6* are expressed in the lung mesenchyme^43^,^44^,^45^,^46^, whereas the others are expressed in the endoderm^1^,^47^. These well-conserved sequences in C-LME indicate that transcription factor binding sites are candidate “trans-factors” that drive the expression of *Tbx4* in the lung mesenchyme during lung development.

Recent studies have shown that the *Tbx4* LME sequence is not present in the actinopterygians and is only found in the sarcopterygians^8^,^9^. Thus, the C-LME sequence has only been conserved in air-breathing vertebrates with lung. There are several air-breathing teleost fish, all of which except the polypterids use a gas bladder^2^. A comparison of the lung and gas bladder reveals that the lung developed from the ventral portion of the foregut endoderm, while the gas bladder developed from the dorsal side^2^,^12^. However, it is not quite this simple, as the gas bladder also expresses the same genes that are expressed during lung development^48^,^49^, including the *Tbx4* genes (M. Okabe, unpublished data), and many fish breathe air using the gas bladder^2^. Therefore, it is difficult to define the lung and gas bladder based on the genes that are expressed and the air-breathing function. However, our findings indicate that it may be possible to separate these organs based on the possession of the C-LME sequence, although additional basal group sequences of Actinopterygii are required to prove this. Fortunately, the genome sequences of spotted gar (*Lepisosteus oculatus*) and Asian arowana (*Scleropages formosus*) have recently been reported^50^,^51^, both of which are air-breathing fish with a gas bladder^2^. Gars are the sister lineage of teleosts and diverged from them before teleost-specific whole genome duplication (three-round whole-genome duplication; 3R-WGD) occurred^17^,^52^,^53^,^54^. In contrast, arowanas belong to the Osteoglossomorpha, which are the most basal group of teleosts after the 3R-WGD^17^. VISTA plots showed that both species seemed to possess the *Tbx4* C-LME, but multiple sequence alignment revealed that many substitutions and deletions/insertions in their C-LME were observed, although only TCF1/LEF1 and GATA6 binding sites were conserved. (see Supplementary Fig. S2a and S2b). Furthermore, the *Tbx4* genomic sequences of both species were much closer to those of other teleosts than those of tetrapods, and several conserved elements were found in gars and teleosts (see Supplementary Fig. S2c). These data imply that the loss of lung in actinopterygians might result from functional loss of C-LME, although the genomes of other basal actinopterygians such as sturgeons should be analyzed.

**Figure 5:**
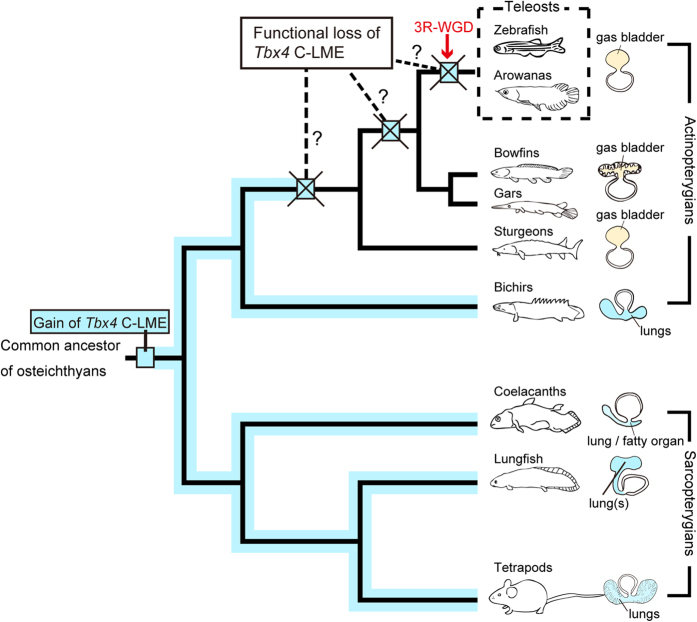
Summary of the *Tbx4* C-LME in osteichthyan evolution. Possession of the *Tbx4* core lung mesenchyme-specific enhancer (C-LME) is summarized on a simplified phylogenetic tree of osteichthyes. The blue line shows the possession of *Tbx4* C-LME in sarcopterygians and actinopterygians, corresponding to the possession of lungs. Lungfish are assumed to possess the *Tbx4* C-LME due to the existence in coelacanths. The blue squares indicate presumptive positions for functional loss of the *Tbx4* C-LME during the evolution of actinopterygians. We indicate the point at which this loss may have occurred. The red arrow indicates the timing of 3R-WGD (teleost specific whole-genome duplication).

This is the first study to demonstrate that the molecular mechanism of lung development in the air-breathing fish bichir is similar to that observed in tetrapods. Our findings also indicate that the molecular mechanism of lung development using the *Tbx4* lung enhancer may already have been present in the common ancestor of the actinopterygians and sarcopterygians (Fig. 5; based on Perry and Sander^14^; Zhu and Yu^36^). Our data show that bichir possesses *Tbx4* C-LME, which is also present in extant sarcopterygians but has been lost in teleosts. This result indicates that unique sarcopterygian specializations are shared osteichthyan characteristics that have been lost in teleosts. Similar recent findings for gar also suggest that unique genome sequences that were present in the sarcopterygians were lost in the teleosts^50^,^55^. This finding is particularly important for improving our understanding of osteichthyan evolution.

Further experiments with *Polypterus* will provide us with great insights into such ancestral osteichthyan characteristics.

## Methods

### Specimens

Male and female adult *P. senegalus* were purchased from a local pet shop (Meito Suien, Japan) and naturally mated. We maintained the fertilized eggs at 28 °C until they reached the required developmental stages^25^, following which we fixed them in 4% paraformaldehyde (PFA) at 4 °C for 2 h or overnight. We recorded the developmental stages in days post fertilization (dpf). All animal experiments were approved by the Animal Care and Experimentation Committee of the Jikei University School of Medicine and were performed in accordance with the approved guidelines.

### Paraffin- and cryosectioning

For paraffin sectioning, we passed the fixed larvae through a series of 70%, 80%, 90%, and 100% ethanol and 100% acetone. They were then embedded in paraffin. We made 4-μm serial sections using a Leica RM2235 microtome and then deparaffinised and stained them with hematoxylin and eosin.

For cryosectioning, we embedded the fixed larvae in optimal cutting temperature compound (Sakura Finetek, Japan) and made 10-μm serial sections using a Leica CM3050S cryostat.

### Cloning and identification of the genes for lung development

To isolate total RNA, we harvested and homogenized 10-day-old larvae using TRIzol Reagent (Life Technologies, CA, USA). The total RNA was then reverse-transcribed using the PrimeScript II 1^st^ strand cDNA Synthesis Kit (TaKaRa Bio, Japan). Primers for the *Fgf10, Nkx2.1, Tbx4*, and *Tbx5* genes were designed based on the genomic sequence of *P. senegalus,* which had been sequenced by our consortium. We performed a polymerase chain reaction (PCR) using Q5 High-Fidelity DNA Polymerase (New England Biolabs, MA, USA) and the following primer sets: *Fgf10* forward, 5′-ATGTGGAAATGGATACTGACA-3′; *Fgf10* reverse, 5′-TCATGCATGGCCCAAAATG-3′; *Nkx2.1* forward, 5′-ATGTCGATGAGCCCCAAGCA-3′; *Nkx2.1* reverse, 5′-TCACCAGGTCCTACCATATAATAGTG-3′; *Tbx4* forward, 5′-ATGCTGCAGGAAAAATCTCCAGCT-3′; *Tbx4* reverse, 5′-TTATTTATCTTCCTTATACCCATCTATCC-3′; *Tbx5* forward, 5′-ATGGCGGACACCGAGGAA-3′; *Tbx5* reverse, 5′-TTAGCTGTTTTCTCCCCATTCCG-3′. Adenine was added to the 3′ ends of the PCR products using Blend Taq Plus (TOYOBO Life Science, Japan), and these were then cloned into the pGEM-T Easy Vector (Promega, WI, USA) and sequenced to identify the full-length sequences of *Nkx2.1*, *Tbx4*, *Tbx5*, and *Fgf10* (DDBJ^56^ accession numbers LC031499, LC031500, LC031501, and LC031502, respectively).

### *In situ* hybridization

Using the plasmids for *Fgf10*, *Nkx2.1*, *Tbx4*, and *Tbx5*, we amplified the inserted DNA using Blend Taq Plus with the M13 primers. The amplified PCR products were then used as templates to synthesize probes for the four genes using Sp6 RNA polymerase. We performed *in situ* hybridization on the sections as previously described^57^.

### Identification of the lung mesenchyme-specific enhancer for the *Tbx4* gene

The LME of *Tbx4* was previously identified by Menke *et al*.^6^. To construct VISTA plots^58^ based on mouse LME, we obtained the genomic sequences for mouse, human, chicken, frog (*X. tropicalis*), coelacanth, zebrafish, and medaka from Ensembl, and used these alongside the genomic sequence of *P. senegalus* (DDBJ^56^ accession number: LC031503), which had been sequenced by our consortium. The binding sites of the transcription factors were then predicted using the rVISTA program^59^ with the TRANSFAC library.

### Functional assay for the core lung mesenchyme-specific enhancer

The *P. senegalus* genome was isolated from a caudal fin clip using the DNeasy Blood & Tissue Kit (Qiagen, Germany), whereas coelacanth *L. chalumnae* genome was obtained from Nikaido *et al*.^8^. We amplified the conserved enhancer region using PrimeSTAR HS DNA Polymerase (TaKaRa Bio, Japan) with two sets of primers for each species: *P. senegalus (ps) Tbx4*C-LME-1 Fwd, 5′-ACGCGTCGACTGACAAATGAACTTCTGAGGAGAACTC-3′; *psTbx4*C-LME-1 Rev, 5′-GGGGTACCCTTATCACCAACGCCAGCCC-3′; *psTbx4*C-LME-2 Fwd, 5′-CCGCCTCGAGCTTATCACCAACGCCAGCCC-3′; *psTbx4*C-LME-2 Rev, 5′-ACGCGTCGACTGAAAAATGAACTACTGATGAGAACTCCT-3′; *L. chalumnae* (*lc*) *Tbx4*C-LME-1 Fwd, 5′-ACGCGTCGACTGAAAAATGAACTACTGAGGAGAACTC-3′; *lcTbx4*C-LME-1 Rev, 5′-GGGGTACCCTTATCACCCACGCTACTCTGC-3′; lc*Tbx4*C-LME-2 Fwd, 5′-GGGGTACCTGAAAAATGAACTACTGAGGAGAACTC-3′; *lcTbx4*C-LME-2 Rev, 5′-CCGCCTCGAGCTTATCACCCACGCTACTCTGC-3′.

The PCR products with the first primer set were cut using *SalI* and *KpnI*, whereas those with the second primer set were cut using *KpnI* and *XhoI*. We then ligated both products into a ptk-EGFP vector to make constructs with 2× enhancers (*psTbx4*C-LME2× and *lcTbx4*C-LME2×). Each plasmid of the *psTbx4*C-LME2× and *lcTbx4*C-LME2× constructs was cut using *SalI* and *SphI*, and the shorter fragment was separated with gel electrophoresis, whereas each of the *psTbx4*C-LME2× and *lcTbx4*C-LME2× plasmids was cut using *XhoI* and *SphI*, and the longer fragment were separated. The shorter and longer fragments were then ligated to each other to make constructs with 4× enhancers (*psTbx4*C-LME4× and *lcTbx4*C-LME4×).

To evaluate the activity of the C-LMEs, the plasmid DNAs were introduced into the lung mesenchyme on the right-hand side of 2-day-old (HH^33^ stage 12) chicken embryos using a CUY21 electroporator (Tokiwa Science, Japan), as previously described^27^. Chicken eggs were purchased from a local supplier (Shiroyama Keien, Japan). The concentration of the plasmid DNA was adjusted to 10–15 μg/μl. The expression vector pCAGGS-Kusabira-orange (1 μg/μl) was co-introduced to monitor the electroporation efficiency. After 45–48 h in culture, we harvested the embryos and fixed them in 4% PFA at 4 °C for 2 h or overnight.

To observe the expression of the transgenes, we made cryosections and immunofluorescently stained them using 1:500 rabbit anti-GFP antibody (MBL, Japan) and 1:500 mouse anti-Kusabira antibody (MBL) as primary antibodies; and 1:500 anti-mouse IgG DyLight 549 (RockLand, PA, USA) and 1:500 anti-rabbit IgG Alexa fluor 488 (Life Technologies) as secondary antibodies. We did not detect any significant difference in the EGFP fluorescent intensity between *psTbx4*C-LME 2× and 4× and between *lcTbx4*C-LME 2× and 4×, and therefore, only present the results for the 4× enhancers.

## Additional Information

**How to cite this article**: Tatsumi, N. *et al*. Molecular developmental mechanism in polypterid fish provides insight into the origin of vertebrate lungs. *Sci. Rep.* **6**, 30580; doi: 10.1038/srep30580 (2016).

## Supplementary Material

Supplementary Information
