# Supplementary material for: Molecular developmental mechanism in polypterid fish provides insight into the origin of vertebrate lungs
Source: Sci Rep. 2016 Jul 28;6:30580. doi: 10.1038/srep30580 (PMC4964569; doi:10.1038/srep30580)
Supplement: Supplementary Information [file srep30580-s1.pdf]

Supplementary information

Supplementary figures (Fig. S1 and S2)

Title

Molecular developmental mechanism in polypterid fish provides insight into the origin of vertebrate lungs

Authors

Norifumi Tatsumi , Ritsuko Kobayashi, Tohru Yano, Masatsugu Noda, Koji Fujimura, Norihiro Okada, Masataka Okabe[*]

Methods

Phylogenetic trees of *P. senegals* identified genes

To verify that each of the four genes (*Fgf10*, *Nkx2.1*, *Tbx4*, and *Tbx5*) were orthologous to those in the model animals, we constructed phylogenetic trees (Supplementary Fig. S1). We obtained the amino acid sequences of these genes in human (*Homo sapiens*), mouse (*Mus musculus*), chicken (*Gallus gallus*), *Xenopus tropicalis*, zebrafish (*Danio rerio*), and medaka (*Oryzias latipes*) from the NCBI, and the sequences for coelacanth (*Latimeria chalumnae*) from Ensembl[59]. The sequences were then aligned for each of the four genes, and the parts with low similarity were removed from each alignment. Following this, maximum likelihood phylogenetic trees were constructed with MEGA6[60] using the best-fit substitution models (*Nkx2.1*, JTT + G; *Fgf10*, LG + G; *Tbx4*, JTT + I; and *Tbx5*, JTT + G), with 10,000 bootstrap repetitions and other default parameters.

Comparative analysis of the *Tbx4* genomic sequence in several species

For VISTA[61] plots, we identified two Asian arowana (*Scleropages formosus*) *Tbx4* sequences (*Tbx4a* and *Tbx4b*, DDBJ[62] accession number: BR001384 and BR001385) from the whole-genome assemblies of Asian arowana[63] in GenBank. Aliment of arowana *Tbx4a* and *Tbx4b* indicated that *Tbx4b* is similar to other teleosts *Tbx4*, therefor we decided to use *Tbx4b* for this analysis. Mouse genome with LME was compared with the obtained the genomic sequences of coelacanth, spotted gar (*Lepisosteus oculatus*)[64], zebrafish, and medaka from Ensembl and the identified bichir genomic sequence (Supplementary Fig. S2a). Aliment of the C-LME conserved regions sequences are shown in Supplementary Fig. S2b.

To obtain more information of C-LME in the actinopterygians and of actinopterygians specific conserved regions, we compared genomic sequences from Ensembl of mouse, gar, arowana, zebrafish, cod (*Gadus morhua*), tilapia (*Oreochromis niloticus*), medaka, stickleback (*Gasterosteus aculeatu*s), tetraodon (*Tetraodon nigroviridis*), and fugu (*Takifugu rubripes*) by VISTA plots (Supplementary Fig. S2c). We used Shuffle-LAGAN[62] for the alignment program in both analysis.

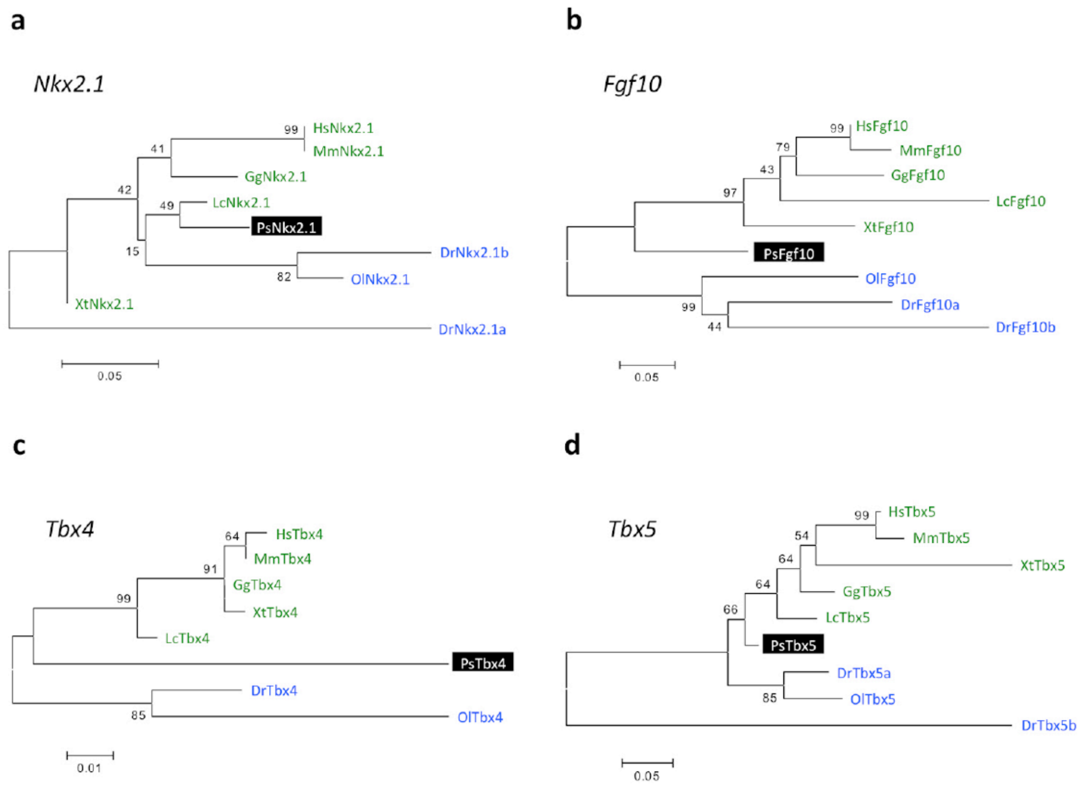

Supplementary Figure S1 | Phylogenetic trees for the four genes that play an important role in early lung development in tetrapods. Phylogenetic trees for (**a**) *Nkx2.1*, (**b**) *Fgf10*, (**c**) *Tbx4*, and (**d**) *Tbx5*. Numbers are bootstrap values for each divergence. Dr, zebrafish *Danio rerio*; Gg, chicken *Gallus gallus*; Hs, human *Homo sapiens*; Lc, coelacanth *Latimeria chalumnae*; Mm, mouse *Mus musculus*; Ol, medaka *Oryzias latipes*; Ps, bichir *Polypteris senegalus*; Xt, frog *Xenopus tropicalis*.

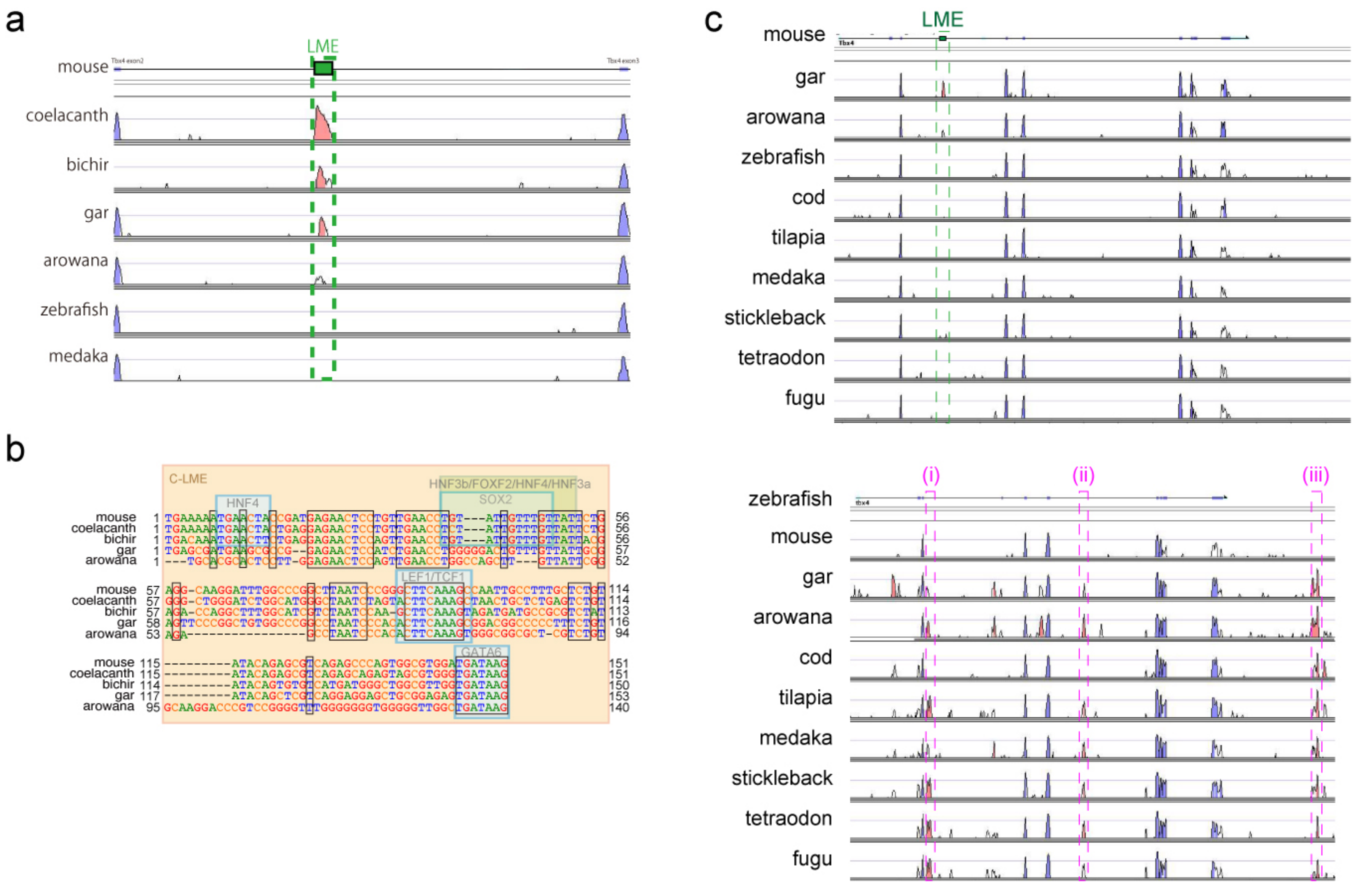
a
LME
mouse
coelacanth
bichir
gar
arowana
zebrafish
medaka
b
C-LME
HNF4
HNF3b/FOXF2/HNF4/HNF3a
SOX2
LEF1/TCF1
GATA6
c
LME
mouse
gar
arowana
zebrafish
cod
tilapia
medaka
stickleback
tetraodon
fugu
(i)
(ii)
(iii)
zebrafish
mouse
gar
arowana
cod
tilapia
medaka
stickleback
tetraodon
fugu

Supplementary Figure S2| Comparative analysis of the *Tbx4* genomic sequence in the actinopterygians.

(a) Mouse LME sequence compared with coelacanth, bichir, gar, arowana, zebrafish and medaka by VISTA plots. (b) LME regions (green box) are shown in the green dot box. Aliment of C-LME conserved region sequence of mouse coelacanth, bichir, gar and arowana. (c and d) Comparative analysis of *Tbx4* genomic regions of mouse, gar, arowana, zebrafish, cod, tilapia, medaka, stickleback, tetraodon and fugu by VISTA plots. (c) Mouse compared with actinopterygians and green dot box showed LME (C-LME) regions. (d) Zebrafish compared with mouse and actinopterygians and three actinopterygians specific conserved peaks (i, ii, and iii) were observed. Only cod did not show the peaks of (i) and (ii) but we observed a conserved sequence in (ii).

59 Herrero, J. *et al.* Ensembl comparative genomics resources. *Database (Oxford)* **2016**, doi:10.1093/database/bav096 (2016).
60 Tamura, K., Stecher, G., Peterson, D., Filipski, A. & Kumar, S. MEGA6: Molecular Evolutionary Genetics Analysis version 6.0. *Mol Biol Evol* **30**, 2725-2729, doi:10.1093/molbev/mst197 (2013).
61 Frazer, K. A., Pachter, L., Poliakov, A., Rubin, E. M. & Dubchak, I. VISTA: computational tools for comparative genomics. *Nucleic Acids Res* **32**, W273-279, doi:10.1093/nar/gkh458 (2004).
62 Mashima, J. *et al.* DNA data bank of Japan (DDBJ) progress report. *Nucleic Acids Res* **44**, D51-57, doi:10.1093/nar/gkv1105 (2016).
63 Bian, C. *et al.* The Asian arowana (*Scleropages formosus*) genome provides new insights into the evolution of an early lineage of teleosts. *Sci Rep* **6**, 24501, doi:10.1038/srep24501 (2016).
64 Braasch, I. *et al.* The spotted gar genome illuminates vertebrate evolution and facilitates human-teleost comparisons. *Nat Genet*, doi:10.1038/ng.3526 (2016).
